# Evaluating the Feasibility and Impact of a Yoga Intervention on Cognition, Physical Function, Physical Activity, and Affective Outcomes in People Living With HIV: Protocol for a Randomized Pilot Trial

**DOI:** 10.2196/13818

**Published:** 2019-05-21

**Authors:** Adria Quigley, Kelly K O'Brien, Marie-Josée Brouillette, Marilyn MacKay-Lyons

**Affiliations:** 1 Department of Physiotherapy Dalhousie University Halifax, NS Canada; 2 Department of Physical Therapy University of Toronto Toronto, ON Canada; 3 Institute of Health Policy, Management and Evaluation University of Toronto Toronto, ON Canada; 4 Rehabilitation Sciences Institute University of Toronto Toronto, ON Canada; 5 Department of Psychiatry McGill University Montreal, QC Canada; 6 Department of Medicine Dalhousie University Halifax, NS Canada; 7 Nova Scotia Health Authority Halifax, NS Canada

**Keywords:** HIV, AIDS, yoga, cognition

## Abstract

**Background:**

Despite lower mortality rates due to combination antiretroviral therapy, people living with HIV (PLWH) are grappling with increasingly complex health issues, including cognitive impairments in areas such as memory, attention, processing speed, and motor function. Yoga has been shown to be an effective form of exercise and mindfulness-based stress reduction for many clinical populations. However, no randomized trials have evaluated the impact of yoga on cognitive and physical function among PLWH.

**Objective:**

The aim of this pilot randomized trial was to determine the feasibility of a yoga intervention to lay the groundwork for a full-scale, multisite, community-based trial for PLWH. Specific objectives are to (1) assess the feasibility of study protocol and procedures, (2) compare cognition in the yoga group with the usual care control group after 12 weeks of the intervention in PLWH, and (3) compare the effects of the 12-week yoga intervention versus control on balance, walking speed, physical activity, mental health, medication adherence, and quality of life among PLWH.

**Methods:**

We propose a pilot randomized trial with 2 parallel groups (yoga versus control). We will recruit 25 PLWH (>35 years) from community and health organizations in Halifax, Canada. After baseline assessment with blinded assessors, participants will be randomly assigned to the yoga or control group, using a random computer generator. Participants in the yoga group will engage in supervised 60-min group-based yoga sessions 3 times a week for 12 weeks at a yoga studio. Participants in the control group will maintain their current physical activity levels throughout the study.

**Results:**

As per the Consolidated Standards of Reporting Trials extension for pilot studies, means of all outcomes, mean change, and 95% CIs will be calculated for each group separately. Two-tailed independent *t* tests and Fisher exact tests will be used to compare groups at baseline. We will analyze quantitative postintervention questionnaire responses using Chi-square tests, and open-ended responses will be analyzed thematically. Intention-to-treat and per-protocol analyses will be used to analyze secondary variables. Changes in outcome variables will be examined between groups and within groups. Effect sizes will be reported for each outcome. A priori adherence and satisfaction criteria will be met if participants attend >70% of the yoga sessions and if >70% of the participants are satisfied with the intervention as determined by a postparticipation questionnaire. Study enrollment began in January 2018, with results expected for October 2019.

**Conclusions:**

This pilot randomized trial will be the first to investigate the feasibility and effect of a yoga intervention on cognitive and physical outcomes among PLWH. This work will inform the feasibility of further investigations in terms of capacity building, participant recruitment and retention, and assessment and intervention protocols.

**Trial Registration:**

ClinicalTrials.gov NCT03071562; https://clinicaltrials.gov/ct2/show/NCT03071562 (Archived by WebCite at http://www.webcitation.org/785sfhWkw)

**International Registered Report Identifier (IRRID):**

DERR1-10.2196/13818

## Introduction

### Cognitive Impairment in People Living With HIV

Despite lower mortality rates due to combination antiretroviral therapy (cART), people living with HIV (PLWH) are grappling with increasingly complex health issues [1], including cognitive impairments in areas such as memory, attention, processing speed, and motor function [2]. Even with the widespread use of cART, 30% to 60% of PLWH experience cognitive impairment [2,3]. Given that the number of people with HIV-associated cognitive impairment is expected to increase 5- to 10-fold by the year 2030 [4], and the incidence of HIV infection is increasing among older adults [5], this issue has become a public health concern [6]. Aging and HIV appear to have combined deleterious effects on both brain structure and function, and some investigators have hypothesized that these effects could be synergistic [7,8]. As such, the combined effect of age and cognitive impairment in HIV has become a concern over the past decade, especially as PLWH now have a life expectancy that rivals that of their HIV-negative counterparts [9]. Proposed mechanisms for cognitive dysfunction include direct attacks of the virus on brain tissue and indirect processes such as local or systemic inflammation [10]. Glial cells, possible reservoirs for the virus, release proinflammatory cytokines and toxins associated with cognitive disorders and neuron degeneration [11]. Protein gp120 damages neurons by causing calcium overload and reducing brain-derived neurotrophic factor, the central growth factor involved in neurogenesis [12].

HIV-associated cognitive impairment has a profound impact on activities of daily living [13], social function [14], quality of life [15], employment [16], and adherence to pharmacological [17] and nonpharmacological treatment [18]. Despite the fact that ~95% adherence to cART is required for adequate viral suppression, 66% of participants in a HIV clinical trial *simply forgot* to take their medications [19]. Pharmacological adherence is a major priority, given that cART is the mainstay of proper HIV management. A study of 267 adults with HIV revealed that those with cognitive impairment performed worse on functional laboratory measures of shopping, cooking, finances, medication management, and work-related skills than those with normal cognition [20]. Furthermore, the authors discovered that poor executive function, learning, attention, working memory, and verbal abilities strongly predicted functional performance [20]. Authors of another study revealed that symptomatic cognitive impairment was associated with significantly worse scores in 8 domains of the Medical Outcomes Survey for HIV (MOS-HIV) [21]. PLWH with cognitive impairment are less likely to be employed [22], have a difficult time returning to work after disability [23], and have difficulties adapting to the demands of work [20].

### Gait and Balance Impairments Among People Living With HIV

Although the cognitive aspects of HIV-associated neurocognitive disorder such as memory, attention, and processing speed have been studied in great detail, the motor aspects have not received much attention. There is evidence of a shared pathology between cognitive and motor functions; a large study of 1549 PLWH revealed a significant relationship between slowed gait and worsening cognitive function [24]. Balance and gait impairments are common among PLWH [25], and they are associated with frailty, higher rates of falls, and increased mortality [26]. Decreased gait speed is linked to higher fall risk, even in those taking cART with undetectable viral loads [26]. A recent systematic review and meta-analysis of 16 cross-sectional studies and 1 prospective cohort study conducted by Berner and colleagues (2017) evaluated the available literature on gait and balance dysfunction in PLWH [27]; a total of 3 [28-30] of 8 studies [25,26,28-33] that examined gait speed reported slowing of fast gait speeds among PLWH compared with controls.

Balance performance tests also reveal balance impairments among PLWH. Using the Single Leg Stance Time Test, Bauer and colleagues (2011) [25] revealed a significant decrease in nonpreferred single leg stance time among obese PLWH compared with seronegative controls in their sample of 86 seropositive and 121 seronegative individuals. Sullivan and colleagues (2011) [34] had similar findings in their sample of 40 female and male PLWH, but they found no differences between groups in tandem stance time. Using the Single Leg Stance Time Test with eyes closed in their sample of 308 PLWH, Tanon and colleagues (2017) [35] determined that 87% of participants demonstrated balance impairments. Performance on the Heel-To-Toe Walk Test with eyes closed [34], the Limits of Stability Test [25,31], and the 360-Degree-Turn Test (among PLWH with obesity only) [25] may also be impaired. Notably, PLWH appear to perform well on the Berg Balance Scale [26,27,36], which indicates that more challenging dynamic balance assessments are required to identify impairments in this population.

### Exercise and Cognitive Function in People Living With HIV

Quigley and colleagues (2018) recently published a scoping review to map the available evidence regarding physical activity and cognitive outcomes (both objective and self-reported) among PLWH [37]. The scoping review included 16 studies: 5 randomized controlled trials (RCTs) [38-42], 3 pre-post single group observational studies [43-45], and 8 cross-sectional studies [46-53], with a total of 1701 PLWH [37]. The noninterventional research indicated a strong association between physical activity levels and cognitive performance as measured by a cognitive battery in PLWH; all 8 cross-sectional studies demonstrated positive associations [46-53]. However, only 2 of the 8 interventional studies—an RCT [41] of aerobic and resistance exercise and a single cohort study involving Tai Chi [43]—revealed positive outcomes regarding cognition in PLWH. McDermott and colleagues [42] conducted the only RCT to directly examine the effect of exercise on an objective measure of cognition in PLWH. Their 16-week aerobic exercise intervention, 3 times per week at 40% to 75% of heart rate reserve neither had an effect on Montreal Cognitive Assessment scores nor had an effect on Trails A and B scores [42]. However, the sample size comprised 11 participants, and the Montreal Cognitive Assessment may not be sensitive to cognitive impairment in PLWH [54]. Clearly, confirmatory evidence of the effect of exercise on cognition in this population is lacking.

### The Effect of Yoga on Cognitive and Physical Function

Yoga has emerged as an effective form of exercise and mindfulness-based stress reduction across many clinical populations [55]. It is an ancient practice combining postures, mindfulness, spirituality, and breath control to enhance flexibility, strength, and balance, and it is increasingly being recognized as a mainstream intervention to promote a more preventative and holistic health care approach [56,57]. Findings of a meta-analysis of 15 RCTs suggest that yoga interventions lasting 1 to 6 months are associated with enhanced overall cognitive function (Hedges  *g*=0.33), attention and processing speed (Hedges  *g*=0.299), executive function (Hedges  *g*=0.27), and memory (Hedges  *g*=0.18) in people with and without chronic diseases [58]. In fact, it appears that acute bouts of yoga may be superior to aerobic exercise for improving inhibition and working memory, as determined by a repeated-measures study of 30 healthy younger women [59]. There are numerous mechanisms thought to underlie cognitive improvements with yoga interventions. It is possible that yoga may contribute to dominance of the parasympathetic nervous system [60,61] while downregulating the sympathetic nervous system and the hypothalamic-pituitary-adrenal axis [61]. A systematic review of 25 RCTs conducted with healthy and chronic disease populations revealed that those who participated in yoga improved their cortisol levels, heart rate, and blood pressure relative to controls [62]. There is also evidence that yoga and other types of mind-body exercise (including Tai Chi) are associated with improved mood; a meta-analysis of 40 interventional studies revealed that Tai Chi has positive effects on both anxiety and depression [63]. Improvements in the stress response with mind-body exercise may contribute to improved cognitive performance [64]; an RCT of 118 older adults revealed that yoga participants had an attenuated cortisol response and improved executive function relative to the control group following an 8-week yoga intervention [64]. Of note, self-reported mood stress and cortisol levels predicted executive function performance [64]. Other potential mechanisms associated with yoga interventions include the learning of novel tasks, which is associated with changes in brain structure and function [58], sustained attention [65], activation of the default mode network (including learning and consolidation functions) [66], and improved meta-cognition (one’s conscious awareness of his or her cognitive processes), which is closely related to executive function [67].

Yoga is also an effective treatment for impaired balance in people with [68-71] and without physical impairments [72,73] because of its positive effects on strength [74], mobility [69], balance self-efficacy [70,71], and visuospatial memory [75]. A 2016 systematic review and meta-analysis of 6 RCTs confirmed that healthy older adults and individuals with various health conditions, such as stroke, Parkinson’s disease, and knee osteoarthritis, reap yoga-induced benefits to postural stability and mobility [76]. The investigators suggested that health care professionals should recommend yoga to older adults as a safe and effective intervention for balance and mobility limitations [76]. There is considerably less research evaluating the effect of yoga on balance, quality of life, and depression in PLWH. In their case-series study of 3 PLWH, Kietrys and colleagues (2018) observed improvements in several gait parameters (including double-limb support time, step length, stride length, stride velocity, and walking velocity) and balance (as measured by the Multidirectional Reach Test) in 2 of the 3 participants following a 4-week yoga intervention [77]. There is some RCT evidence for the benefits of yoga on quality of life [78] and depression [79] in PLWH; however, the former study did not involve yoga postures, and the latter intervention was only a month in total duration. To date, no RCTs have evaluated the impact of yoga on cognitive and physical performance among PLWH.

### Purpose and Objectives

The purpose of this pilot RCT is to determine the feasibility of a yoga intervention to lay the groundwork for a full-scale, multisite, and community-based trial with PLWH. Specific objectives are to (1) assess the feasibility of the study protocol and procedures, (2) compare cognitive function in PLWH in a yoga intervention group with a usual care control group among PLWH after 12 weeks of the intervention, and (3) compare the effects of the 12-week yoga intervention versus control on balance, walking speed, physical activity, mental health, medication adherence, and quality of life in PLWH.

## Methods

### Design

**Figure 1:**
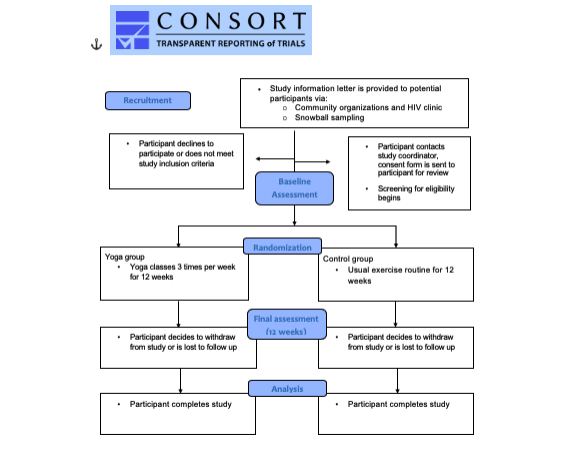
Consolidated Standards of Reporting Trials flow diagram.

We propose a pilot randomized trial with 2 parallel groups, comparing the yoga group with a usual care control group using quantitative methods of data collection. Figure 1 outlines the sequencing of the study protocol. The conceptual framework for pilot and feasibility studies created by Eldridge and colleagues [80] and the Consolidated Standards of Reporting Trials (CONSORT) 26-item checklist for randomized pilot and feasibility studies will be employed to ensure methods are properly defined and reported [81]. The study is guided by a community advisory committee comprising 7 members of the HIV community and 3 representatives from local HIV organizations. Our research team held consultations with the community advisory committee to assist with study design and recruitment strategy.

### Participants

We will identify cognitive concerns on the Communicating Cognitive Concerns Questionnaire (C3Q) with a cut-off of 35 points or less [82]; in addition, we will include a maximum total of 25 PLWH who are aged 35 years or older of any gender, are English speaking, live within 50 km of the study site, are able to provide informed consent, and are deemed medically stable as assessed by the Physical Activity Readiness Questionnaire Plus [83]. Study exclusion will include regular participation in a yoga program during the 6 months before study commencement.

### Recruitment

Recruitment will occur via newsletters and posters at community organizations and health centers in Halifax, Nova Scotia. Furthermore, staff at the local HIV Clinic have agreed to approach eligible individuals and provide them with a study information brochure. To obtain a sample that is diverse in terms of ethnicity, gender, and severity of HIV disease, we will also employ snowball sampling techniques, whereby potential participants will be asked to identify other potential participants. All interested individuals will contact the study coordinator. The coordinator will explain the general purpose and procedures of the study, risks and potential benefits, time commitment, and responsibilities of the participants. Each potential participant will be informed that health care services will not be affected by study participation or withdrawal. A copy of the consent form will be provided and reviewed, and all the questions will be answered to the potential participant’s satisfaction. Potential participants who remain interested in enrolling in the study will be asked to sign the consent form approved by the local Research Ethics Board (REB).

### Randomization

After baseline assessment, an individual not directly involved in the study will randomly assign participants in a 1:1 ratio to the yoga or control group using a random computer generator. Group assignment of each participant will be concealed in individual opaque envelopes that will remain sealed until after completion of the baseline assessment. The number of participants screened and randomized to each group will be recorded, as per the CONSORT extension for randomized pilot trials [81].

### Ethical Considerations

The study protocol was approved by the REB (protocol reference #1022158). The procedures will be followed in accordance with institutional ethical standards and the Helsinki Declaration. The trial was registered on ClinicalTrials.gov. Proposed amendments to the protocol will be submitted for review to the REB. For ethical reasons, we cannot ask participants to avoid making medication changes; any changes participants make to their medications will be documented. Unanticipated or adverse events will be reported immediately to the REB. Participant confidentiality and autonomy will be maintained throughout the study, and data will be anonymized and secured. Study data will be stored in a locked office at Dalhousie University. Electronic data will be stored in encrypted form and will exclusively be accessed by the research team. Restricting access to data on-site until the data have been appropriately coded and deidentified will mitigate the risk of residual disclosure. All data will be destroyed after 7 years. Decisions to stop participating will be respected. To offset participants’ personal and travel costs, we will provide bus tickets for assessments and yoga sessions, and we will provide parking reimbursement, snacks, and honoraria for the assessments.

### Intervention Protocols

#### Yoga Group

Groups of 4 to 5 participants will engage in 60-min group-based Hatha-style yoga sessions 3 times per week for 12 weeks under the supervision of a yoga-certified physiotherapist at a local yoga studio. Classes will begin with a 15-min warm-up, which includes seated meditation, breathing exercises, shoulder and neck stretches, back mobility exercises, and sun salutations. Then, participants will perform 10 min of standing and 15 min of balance poses, followed by 10 min of abdominal work and back-bends. The class will finish with 5 min of final rest (savasana). The yoga protocol can be seen in Table 1.

**Table 1:** Yoga protocol.

Warm-up (15 min)	Standing poses (10 min)	Balance poses (15 min)	Abdominals and back bends (10 min)	Cool down (10 min)
Seated meditation; alternate nostril breathing; bellows breath; shoulder/neck stretches; cat-cow forward fold; sun salutations	Warrior 1; warrior 2; triangle; extended side angle; reverse warrior; high lunge with twist	Tree pose; eagle pose; standing holding knee; modified warrior 3 (chair support); half moon	Bird-dog; side plank; bridge; cobra; sphinx	Twist; cobbler’s pose; hip stretches; corpse pose; side-lying; seated om

#### Yoga Protocol

Yoga mats, blocks, chairs, and straps will be provided to the participants. Postures will be modified for people with balance impairments or neuropathies. If participants are unable to get down to the floor or balance without support, postures will be performed with the use of a chair or other props. As Indigenous people are overrepresented in the HIV epidemic in Canada (they represented 11.3% of all new infections in 2016) [84], the sample population should reflect the cultural diversity within the catchment area of the study. Every month, a smudging ceremony with an Elder representing the Indigenous people will take place for 5 to 10 min before class commencement. The rationale for performing the smudging ceremony is that it is commonly associated with yoga practices [85]; in fact, a recent survey of 360 yoga practitioners identified spirituality as a common reason for starting and maintaining their yoga practice [86].

#### Attendance Policy

Of the total of 36 sessions (3 classes a week for 12 weeks), each participant will be encouraged to attend 70% of classes. Consideration will be given to withdrawing a participant from the study if the participant cancels or does not attend more than 6 sessions for reasons other than illness. In the event of a reversible illness that results in the participant being absent for more than 6 sessions, the participant will be withdrawn from the study and offered to be reenrolled in the yoga group after an 8-week washout period. If a session is cancelled, a make-up session will be scheduled.

#### Control Group

The control group will be asked to continue with its regular exercise routine, and the group will be asked to not make any changes during the study. Interested participants in the control group will be offered the opportunity to attend ongoing yoga classes as frequently as they would like, following study completion.

#### Assessment Protocol

As per the CONSORT extension for pilot RCTs, the number of participants screened for eligibility, randomly assigned, received intended treatment, and assessed for each objective will be recorded [81]. Study data will be collected in the Physiotherapy department at Dalhousie University and managed using Research Electronic Data Capture (REDCap) software (REDCap Inc) [87]. The authors will provide access to the study’s REDCap (REDCap Inc) data upon request. Table 2 outlines the outcome variables and measurement tools.

**Table 2:** Outcomes and measurement tools.

Outcome	Measurement tool	Objective measure or self-report
Yoga readiness (previous experience with yoga, injuries and ability level)	Yoga readiness questionnaire (yoga group only)	Self-report
Cognitive performance	Communicating Cognitive Concerns questionnaire; Brief Cognitive Ability Measure	Self-report; objective
Motor function (balance, walking speed)	Community Mobility and Balance Scale; 10-meter walk test	Objective; objective
Mental health	Hospital Anxiety and Depression Scale	Self-report
Quality of life	Medical Outcomes Survey	Self-report
Medication adherence	Simplified Medication Adherence Questionnaire	Self-report
Physical activity	Rapid Assessment of Physical Activity; Fitbit Flex 2—total distance walked (km) and steps taken per day	Self-report; objective
Participant satisfaction, safety, comfort, fatigue, and benefits	Postparticipation survey (yoga group only)	Self-report

### Outcome Variables and Measurement Tools

#### Demographic Information

We will administer a 13-item paper-based self-reported questionnaire asking about age, sex, gender, ethnicity, education level, employment, income, comorbidities, year diagnosed with HIV, viral load (if known), CD4 count (if known), medications, comorbidities, and physical activity (how often the participant was physically active in the previous week) at baseline to describe the sample and assess group comparability. Participants randomized to the yoga group will be asked to fill out a yoga-readiness questionnaire we created to provide the yoga instructor with safety and injury information.

##### Primary Measures

Many domains of feasibility will be assessed by both participants and study personnel using monitoring processes and a 13-item paper-based post-intervention questionnaire, which includes both questions on a Likert scale ranging from strongly disagree to strongly agree and open-ended questions (see Multimedia Appendix 1):

1. Project coordination (team building, communication and meetings, collaboration, consensus building, troubleshooting, scheduling, protocol consistency, and timelines). Any issues with (or changes to) the study protocol or scheduling will be documented.
2. Participant issues (recruitment, comfort, satisfaction, safety, attendance, time commitment, attrition, and reasons for ineligibility drop out/declining to participate), as assessed by the postintervention questionnaire and documentation by the study coordinator.
3. Assessment protocol elements (time and personnel requirements, usefulness of outcome variables, participant burden, and feasibility) will be recorded by the study coordinator.
4. Intervention protocols (time, equipment, and personnel requirements) will be recorded by the study coordinator.
5. Data quality (completeness, intra/interparticipant variability, interpretability, and trends) will be checked by the study coordinator. Per the CONSORT checklist, our a priori adherence and satisfaction criteria will be met if participants attend 70% of the yoga sessions and if 70% of the participants are satisfied with the yoga intervention as per the postparticipation questionnaire.

##### Secondary and Tertiary Measures

Cognition, physical performance (balance, walking speed), physical activity, and affective (mental health, quality of life, and medication adherence) evaluations will be administered at baseline and postintervention (12 weeks) by a trained assessor, blinded to the group assignment. The rationale for blinding the assessor is to reduce bias in scoring during the assessment sessions. The estimated length of time for the assessment sessions is 2 hours per participant. We will measure *cognitive function* using the Brief Cognitive Ability Measure (B-CAM), a computerized cognitive test developed for PLWH, using Rasch measurement theory and analysis that takes 30 min to administer [88,89]. The B-CAM provides a measure of global cognition that is calibrated—the intervals between logits are equal, meaning the data are continuous [88,90].

Cognitive domains tested with the B-CAM include visual detection (reaction time), Flanker task (response inhibition) [91], memory (learning and recall of 8 words), Shape 2-back (working memory) [92], Corsi block-tapping forward and back tests (visuospatial memory) [93], verbal fluency (letters F-A-S in English) mini Trail Making Test B (executive function) [94], and the Tower of London test (planning) [95]. The scoring of the B-CAM ranges from 0 to 24, with higher values indicating better global cognition [90]. To reduce the likelihood of practice effects, different versions of the B-CAM are performed at baseline and final assessments [90]. Group-based trajectory analysis has revealed that no practice effects were found at the item level [90].

Self-reported cognition will also be assessed using the C3Q, an 18-item paper-based questionnaire that was developed to estimate the presence and frequency of memory, attention, executive function, visuospatial, speech and language, behavior and emotion, and cognitive challenges among PLWH [82]. The frequency of such challenges are recorded by the participant on a 3-point scale: frequently (almost every day), sometimes (once a week), or rarely (once a month) [82].

*Balance* will be measured using the Community Balance and Mobility (CB&M) test, a high-level balance assessment of tasks performed in the community, developed for people with traumatic brain injury [96]. It is a valid and reliable measure of dynamic postural control in people with traumatic brain injury [96,97] and older community-dwelling individuals [98], and it is not as susceptible to ceiling effects as the Berg Balance Scale [98,99]. *Walking speed* will be measured using the 10-meter walk test because of the association of gait speed with cognitive performance in PLWH [24], its previous use in the HIV literature [26], and its ability to predict survival in older adults [100]. *Depression* will be assessed using the Hospital Anxiety and Depression Scale, a paper-based self-report questionnaire [101], which has very good to excellent internal consistency, test-retest reliability and convergent validity, and acceptable discriminant validity in PLWH [102]. Quality of life will be assessed using MOS-HIV, a paper-based questionnaire that comprises 10 domains (physical function, social function, role function, cognitive function, pain, mental health, energy, health distress, quality of life, and overall health), with good to high internal consistency and construct validity in PLWH [103]. *Physical activity* will be assessed using the Rapid Assessment of Physical Activity, a 9-item paper-based questionnaire that measures moderate and vigorous physical activity, including strength and flexibility within the last week [104]. It was validated in older adults [104], and it has been used in studies with people with HIV [105]. Objective levels of physical activity (total distance walked, and number of steps taken per day) will be measured using accelerometers (Fitbit flex 2) [106]. Accelerometer data will be electronically synced and downloaded after weeks 1 and 12 and stored in an encrypted file. Participants will also be asked about *Medication adherence* (specifically cART), measured with the paper-based Simplified Medication Adherence Questionnaire (SMAQ), which has 72% sensitivity, 91% specificity, and a likelihood ratio of 7.94 for nonadherent patients [107].

### Participant Safety

Participants will be monitored throughout the yoga sessions and the assessments. If a participant presents with any medical or safety concerns, the supervising physiotherapist will provide the appropriate first aid or injury treatment; then, the supervising physiotherapist will refer the participant to the participant’s family physician for follow-up. Any harms or unanticipated effects will be recorded as per the CONSORT checklist [81]. Owing to the low-risk nature of the study, we do not anticipate any additional safety or medical issues associated with the yoga interventions.

## Results

### Data Analysis

All questionnaires and measures will be assessed for missing data. The data will be analyzed to determine if the assumptions for parametric tests are met. Descriptive statistics will be used to characterize the participants. As per the CONSORT extension for pilot studies, means of all outcomes, mean change, and 95% CIs will be calculated for each group separately. We will also follow the Sex and Gender Equity in Research guidelines [108] by disaggregating data by sex and gender. Participant dropouts will also be reported disaggregated by sex.

Independent *t* tests and Fisher exact tests will be used to compare the 2 groups at baseline. If the 2 groups differ at baseline, that variable will be included in the analysis as a covariate. We will analyze quantitative postintervention questionnaire responses using Chi-square tests, and open-ended responses will be analyzed thematically. Intention-to-treat and per-protocol analyses will be used in the analysis of the secondary variables. Changes in outcome variables will be examined between groups and within groups. Floor and ceiling effects will be calculated for the CB&M test. Effect sizes will be reported for each outcome. Alpha level will be set at .05, using 2-tailed for all inferences, and data will be analyzed with SPSS Version 25 (SPSS Inc). As this is a pilot study, sample size calculations are not recommended [81]. This pilot study will not be adequately powered to conclusively state the influence of the intervention on study outcomes, but if trends are promising, a future, more adequately powered trial will be planned. This pilot study will provide preliminary data for future sample size calculations.

Study enrollment began in January 2018, with results expected in October 2019.

### Dissemination

Study results will be disseminated to PLWH, researchers, health care providers, community-based organizations, stakeholders, and policy makers. Knowledge translation will take place via peer-reviewed journals, podium and poster presentations at conferences and forums, newsletters, and presentations at community-based organizations.

## Discussion

### Study Strengths

This pilot implementation trial will be the first to investigate the effect and feasibility of a yoga intervention on cognitive and physical outcomes in PLWH. Not only will the study generate preliminary data about the effects of yoga on cognitive and physical function, but it will also inform the feasibility and utility of further investigation in terms of team capacity building, recruitment and retention strategies, and assessment of intervention protocols. The focus of the project is clearly aligned with a key research priority of the Canada-International HIV and Rehabilitation Research Collaborative, which is to determine the effectiveness of rehabilitation interventions and service delivery models [109].

Our research addresses HIV beyond a biological perspective to reduce not only physical limitations but also the social impact of HIV. By targeting an inexpensive nonpharmacological intervention, we hope to identify feasible community-based strategies that may contribute to slowing the health-related consequences of HIV while improving quality of life for PLWH.

### Anticipated Challenges and Limitations

Potential challenges will include recruitment and retention of participants over the course of the 12-week intervention. With approximately 500 PLWH living in the local area [110], we anticipate that by involving community leaders and end users from the outset of conceptualization and planning and conducting the study in a familiar community setting, we will successfully recruit 25 PLWH. Although attrition is of concern in exercise studies requiring multiple visits, a 2015 study on yoga and meditation reported an overall attendance rate of 89% among PLWH [111].

Study limitations include a lack of mechanism to confirm HIV diagnoses for participants not recruited from the HIV clinic and limited study inclusion to individuals who speak and understand English, which may reduce the generalizability of our findings. Participants were also not asked about substance abuse or specific comorbidities, such as peripheral neuropathy, which may affect cognitive and physical performance.

## Appendix

Multimedia Appendix 1 Postparticipation questionnaire.

Multimedia Appendix 2 Canadian Institutes of Health Research peer-review reports.

## Abbreviations

B-CAM: Brief Cognitive Ability Measure
C3Q: Communicating Cognitive Concerns Questionnaire
cART: combination antiretroviral therapy
CB&M: Community Balance and Mobility
CONSORT: Consolidated Standards of Reporting Trials
MOS-HIV: Medical Outcomes Survey for HIV
PLWH: people living with HIV
RCT: randomized controlled trial
REB: Research Ethics Board
REDCap: Research Electronic Data Capture
