# Supplementary material for: Evaluating the Feasibility and Impact of a Yoga Intervention on Cognition, Physical Function, Physical Activity, and Affective Outcomes in People Living With HIV: Protocol for a Randomized Pilot Trial
Source: JMIR Res Protoc. 2019 May 21;8(5):e13818. doi: 10.2196/13818 (PMC6547772; doi:10.2196/13818)
Supplement: Multimedia Appendix 1 [file resprot_v8i5e13818_app1.pdf]

EXERCISE SESSIONS

Please check the box (√) that indicates your level of agreement with each of the following statements.

<u>Safety</u>: I felt safe during the exercise sessions.

☐ Strongly disagree	☐ Disagree	☐ Neutral	☐ Agree	☐ Strongly agree

Comments:

<u>Fatigue</u>: I felt fatigued by the end of each exercise session.

☐ Strongly disagree	☐ Disagree	☐ Neutral	☐ Agree	☐ Strongly agree

Comments:

<u>Comfort</u>: I felt comfortable during the exercise sessions.

☐ Strongly disagree	☐ Disagree	☐ Neutral	☐ Agree	☐ Strongly agree

Comments:

<u>Progression</u>: The difficulty of the exercise sessions over the 24-week study was increased appropriately.

☐ Strongly disagree	☐ Disagree	☐ Neutral	☐ Agree	☐ Strongly agree

Comments:

<u>Enjoyment</u>: I enjoyed the exercise sessions.

☐ Strongly disagree	☐ Disagree	☐ Neutral	☐ Agree	☐ Strongly agree

Comments:

<u>Motivation</u>: I was motivated to work hard during the exercise sessions.

☐ Strongly disagree	☐ Disagree	☐ Neutral	☐ Agree	☐ Strongly agree

Comments:

<u>Benefits</u>: In the end, I feel the exercise sessions were beneficial.

☐ Strongly disagree	☐ Disagree	☐ Neutral	☐ Agree	☐ Strongly agree

Comments:

GENERAL QUESTIONS ABOUT THE PROJECT

1. Have you noticed anything in your day-to-day life that you feel has been affected by participating in this project?

2. Is there anything that you feel has been particularly helpful about having participated in this project?

3. Is there anything that has been disappointing about participating in this project?

4. Have you noticed any changes in your thinking ability since participating in this project? If so, describe the change(s)?

5. Have you noticed any changes in the quality or length of your sleep since participating in this project? If so, describe the change(s)?

6. Any additional comments?
