# Supplementary material for: Evaluating the Feasibility and Impact of a Yoga Intervention on Cognition, Physical Function, Physical Activity, and Affective Outcomes in People Living With HIV: Protocol for a Randomized Pilot Trial
Source: JMIR Res Protoc. 2019 May 21;8(5):e13818. doi: 10.2196/13818 (PMC6547772; doi:10.2196/13818)
Supplement: Multimedia Appendix 2 [file resprot_v8i5e13818_app2.pdf]

**Application Number / Numéro de demande:**	371452
**Name of Applicant / Nom du chercheur:**	Mackay-Lyons, Marilyn Joan
**Review Type / Type d'évaluation:**	Committee Member 1/Membre de comité 1
**Competition:**	2016-06-28 Catalyst Grant: HIV/AIDS Community Based Research
**Concours:**	2016-06-28 Subvention catalyseur : recherche communautaire sur le VIH/sida
**Committee:**	HIV/AIDS Community-Based Research (merged)
**Comité:**	Recherche communautaire sur le VIH/SIDA (fusionné)

Potential Impact

Comments:

371452 Comparing the effects of two community-based exercise programs on cognition , balance, mobility and quality of life of older people living with HIV/AIDS: A pilot randomized controlled trial.

The purpose of this study is to examine the effects of exercise on the cognition of older people living with HIV. The long term goal is to increase access to community-based exercise interventions shown to enhance cognitive health PHAs. The pilot will compare the effects of two 12 week exercise programs (yoga and fitness training) on the quality of life of HIV positive individuals over 50(n=20). Gender considerations have been taken into account for this proposal. The study will take place in Halifax

The research team consists of diverse individuals with many years of experience in their areas of expertise such as HIV and Aboriginal community leadership, knowledge use, and research. The program will include an Aboriginal Elder who will offer a cultural context for the study.

This proposal sets forth an innovative idea and original premise, which is supported by the literature review (yoga intervention on cognitive outcomes with PHAs). The preliminary pilot will generate data about effects of exercise on the cognitive impairments of PHAs and inform the feasibility of further investigation. The outcome intents to provide health care professionals with strategies beyond the biological perspective.

The design utilizes a single –blinded randomized controlled trial, two randomly assigned intervention groups, which will incorporate Elders, prayer and smudging. Community engagement is emphasized involving numerous community organizations in Nova Scotia and is evidenced by partner support letters.

The budget is practical.

Knowledge translation will include a national partner, CWGHR which will disseminate the study results to PHAs, policy makers, and health professionals through social media, websites, and interprofessional learning curricula.

Potential impact 4.0
Scientific Merit 3.6

**Application Number / Numéro de demande:**	371452
**Name of Applicant / Nom du chercheur:**	Mackay-Lyons, Marilyn Joan
**Review Type / Type d'évaluation:**	Committee Member 1/Membre de comité 1
**Competition:**	2016-06-28 Catalyst Grant: HIV/AIDS Community Based Research
**Concours:**	2016-06-28 Subvention catalyseur : recherche communautaire sur le VIH/sida
**Committee:**	HIV/AIDS Community-Based Research (merged)
**Comité:**	Recherche communautaire sur le VIH/SIDA (fusionné)

Scientific Merit

Comments:

371452 Comparing the effects of two community-based exercise programs on cognition , balance, mobility and quality of life of older people living with HIV/AIDS: Apilot randomized controlled trial.

The purpose of this study is to examine the effects of exercise on the cognition of older people living with HIV. The long term goal is to increase access to community-based exercise interventions shown to enhance cognitive health PHAs. The pilot will compare the effects of two 12 week exercise programs (yoga and fitness training) on the quality of life of HIV positive individuals over 50(n=20). Gender considerations have been taken into account for this proposal. The study will take place in Halifax

The research team consists of diverse individuals with many years of experience in their areas of expertise such as HIV and Aboriginal community leadership, knowledge use, and research. The program will include an Aboriginal Elder who will offer a cultural context for the study.

This proposal sets forth an innovative idea and original premise, which is supported by the literature review (yoga intervention on cognitive outcomes with PHAs). The preliminary pilot will generate data about effects of exercise on the cognitive impairments of PHAs and inform the feasibility of further investigation. The outcome intents to provide health care professionals with strategies beyond the biological perspective.

The design utilizes a single –blinded randomized controlled trial, two randomly assigned intervention groups, which will incorporate Elders, prayer and smudging. Community engagement is emphasized involving numerous community organizations in Nova Scotia and is evidenced by partner support letters.

The budget is practical.

Knowledge translation will include a national partner, CWGHR which will disseminate the study results to PHAs, policy makers, and health professionals through social media, websites, and interprofessional learning curricula.

Potential impact 4.0
Scientific Merit 3.6

**Application Number / Numéro de demande:**	371452
**Name of Applicant / Nom du chercheur:**	Mackay-Lyons, Marilyn Joan
**Review Type / Type d'évaluation:**	Committee Member 2/Membre de comité 2
**Competition:**	2016-06-28 Catalyst Grant: HIV/AIDS Community Based Research
**Concours:**	2016-06-28 Subvention catalyseur : recherche communautaire sur le VIH/sida
**Committee:**	HIV/AIDS Community-Based Research (merged)
**Comité:**	Recherche communautaire sur le VIH/SIDA (fusionné)

Potential Impact

Comments:

This comparative study has excellent potential impact on cognitive and physical health outcomes for older people living with HIV/AIDS. The comprehensive literature review indicates that greater than 50% of PHAs live sedentary lifestyles and suffer increasing cognitive impairments as the disease progresses, while the literature shows a strong correlation between exercise and cognition. The comparative aspect will produce interesting results that can be taken up by community and health organizations working with PHAs. The study promises to be a strong contribution to the research in this area and to the HIV/AIDS health literature. The outcomes may be readily transferable to other health areas. The community based and Indigenous inclusion bodes well for knowledge translation and mobilization activities.

**Application Number / Numéro de demande:**	371452
**Name of Applicant / Nom du chercheur:**	Mackay-Lyons, Marilyn Joan
**Review Type / Type d'évaluation:**	Committee Member 2/Membre de comité 2
**Competition:**	2016-06-28 Catalyst Grant: HIV/AIDS Community Based Research
**Concours:**	2016-06-28 Subvention catalyseur : recherche communautaire sur le VIH/sida
**Committee:**	HIV/AIDS Community-Based Research (merged)
**Comité:**	Recherche communautaire sur le VIH/SIDA (fusionné)

Scientific Merit

Comments:

This pilot project is randomized control trial to assess the effects of two 12-week, randomly assigned, community-based, exercise programs - yoga and standard fitness training (strengthening and aerobic exercise) - on cognition, balance, walking, mental health and quality of life in 20 people >50 years of age living with HIV in the Halifax area. Each week an Aboriginal elder will offer the participants an opening prayer and a smudging ceremony at the start of the exercise session. The team has selected Yoga as one of the interventions due to its physical but also spiritual, emotional, and mental dimensions and the potential for it to help stave off some of the devastating consequences of the HIV infection. The primary hypothesis is that the effect of yoga-mindfulness on enhancing cognition will be greater than that of standard exercise.

The long-term goal of the study is to increase access to community-based exercise interventions shown to enhance cognitive health among PHAs. The main purpose of this pilot study is to compare the effects of yoga versus a standard exercise program for PHAs with impaired cognition to lay the groundwork for a full-scale, multi-site, community-based trial. The specific objectives are:
1) To compare the effect of yoga versus a standard exercise program on cognition in PHAs with cognitive impairments (primary objective).
2) To assess feasibility of study protocols and procedures (secondary objective).
3) To compare effects of yoga versus a standard exercise program on balance, walking speed, mental health and QoL (tertiary objective).

The team complement is stellar with several outstanding researchers in related fields. The knowledge user complement is strong. This bodes exceptionally well for the feasibility of meeting the project goals and objectives. The methodology is sound and clearly explained. Indigenous community support and engagement are strong with an array of good letters of support. Baseline data will be gathered on multiple domains including cognitive functioning and depression levels, followed by inferential analyses.

The proposal outlines the study in detail and includes sample intervention activities agendas. The timeline and team roles/activities are clear and enhance the feasibiity of the project. The community based research elements are explicitly outlined. The budget is detailed with small in-kind contributions. There are no budget concerns.Gender and sex issues will be addressed as part of the analysis. The team intends to extend the study by conducting a full-scale, multi-site investigation.

This is a meritorious study and team with high potential for scientific merit.

Rating of Scientific Merit 4.30

Rating of Potential Impact 4.39
